# Supplementary material for: Acute Coronary Syndrome, Stroke, and Mortality after Community-Acquired Pneumonia: Systematic Review and Meta-Analysis
Source: J Clin Med. 2023 Mar 29;12(7):2577. doi: 10.3390/jcm12072577 (PMC10095577; doi:10.3390/jcm12072577)
Supplement: Supplementary file 1 [file jcm-12-02577-s001.zip › jcm-2242972-supplementary.pdf]

S1 – Search strategy

**1. MEDLINE VIA PUBMED**		Results	Date
#1	"Pneumonia"[Mesh]	275,527	11/August/2022
#2	"Respiratory Tract Infections"[Mesh]	553,998	
#3	"Acute Coronary Syndrome"[Mesh]	19,000	
#4	"Stroke"[Mesh]	161,982	
#5	"Mortality"[Mesh]	419,169	
#6	#1 OR #2 AND #3 AND #4 AND #5	21	

**2. GOOGLE SCHOLAR**		Results	Date
#1	All in title "pneumonia"	384,000	11/August/2022
#2	All in title "respiratory tract infections"	33,100	
#3	All in title "acute coronary syndrome"	51,600	
#4	All in title "stroke"	2,570,000	
#5	All in title "mortality"	2,670,000	
#6	#1 OR #2 AND #3 AND #4 AND #5	342	

**3. SCOPUS**		Results	Date
#1	TITLE-ABS-KEY ('pneumonia')	371,143	11/August/2022
#2	TITLE-ABS-KEY ('respiratory tract infections')	160,073	
#3	TITLE-ABS-KEY ('acute coronary syndrome')	72,527	
#4	TITLE-ABS-KEY ('stroke')	491,166	
#5	TITLE-ABS-KEY ('mortality')	1,670,811	
#6	#1 OR #2 AND #3 AND #4 AND #5	130	

**4. EMBASE**		Results	Date
#1	'pneumonia'/exp	394,378	11/August/2022
#2	'lower respiratory tract infection'/exp	318,929	
#3	'acute coronary syndrome'/exp	69,281	
#4	'cerebrovascular accident'/exp	396,757	
#5	'mortality'/exp	1,327,290	
#6	(#1 OR #2) AND #3 AND #4 AND #5	125	
#7	#1 AND #2 AND #3 AND #4 AND #5	26	

**5. WEB OF SCIENCE**		Results	Date
#1	"pneumonia" (Title)	48,400	11/August/2022
#2	"respiratory tract infection" (Title)	6,158	
#3	"acute coronary syndrome" (Title)	25,259	
#4	"stroke" (Title)	161,838	
#5	"mortality" (Title)	178,994	
#6	#1 AND #2 AND #3 AND #4 AND #5	13	

**6. SCIENCEDIRECT**		Results	Date
#1	"pneumonia"	10,211	11/August/2022
#2	"respiratory tract infection"	67,026	
#3	"acute coronary syndrome"	3542	
#4	"stroke"	144,179	
#5	"mortality"	138,913	
#6	#1 AND #2 AND #3 AND #4 AND #5	9	

S2 - General characteristics of the studies excluded.

Reference number in the main text	Country/Region of publication	Study	Reason for exclusion
	USA	Corrales-Medina VF, Musher DM, Wells GA, Chirinos JA, Chen L, Fine MJ. Cardiac complications in patients with community-acquired pneumonia: incidence, timing, risk factors, and association with short-term mortality. Circulation. 2012;125(6):773-81. doi: 10.1161/CIRCULATIONAHA.111.040766.	No control group (without CAP).
	Canada	Johnstone J, Eurich DT, Majumdar SR, Jin Y, Marrie TJ. Long-term morbidity and mortality after hospitalization with community-acquired pneumonia: a population-based cohort study. Medicine (Baltimore). 2008;87(6):329-334. doi: 10.1097/MD.0b013e318190f444.	No control group (without CAP).
	Spain	Viasus D, Garcia-Vidal C, Manresa F, Dorca J, Gudiol F, Carratalà J. Risk stratification and prognosis of acute cardiac events in hospitalized adults with community-acquired pneumonia. J Infect. 2013;66(1):27-33. doi: 10.1016/j.jinf.2012.09.003.	No control group (without CAP).
	Peru	Lazo BD. Complicaciones cardiovasculares en pacientes hospitalizados por neumonía en el Hospital Casimiro Ulloa-2014 [Physician degree thesis]. Lima, Peru: Faculty of human medicine, Ricardo Palms University; 2016 [accessed on 1 February 2023]. Available from: https://repositorio.urp.edu.pe/handle/20.500.14138/539	No control group (without CAP).
	USA	Musher DM, Rueda AM, Kaka AS, Mapara SM. The association between pneumococcal pneumonia and acute cardiac events. Clin Infect Dis. 2007;45(2):158-65. doi: 10.1086/518849.	No control group (without CAP).
	USA	Ramirez J, Aliberti S, Mirsaeidi M, Peyrani P, Filardo G, Amir A, et al. Acute myocardial infarction in hospitalized patients with community-acquired pneumonia. Clin Infect Dis. 2008;47(2):182-7. doi: 10.1086/589246.	No control group (without CAP).
	USA	Mortensen EM, Coley CM, Singer DE, Marrie TJ, Obrosky DS, Kapoor WN, et al. Causes of death for patients with community-acquired pneumonia: results from the Pneumonia Patient Outcomes Research Team cohort study. Arch Intern Med. 2002;162(9):1059-64. doi: 10.1001/archinte.162.9.1059.	No control group (without CAP).
	Spain	Garcia FJA, Poyo MJ, Juanes DI, Begoña LMM, Bello MMC, Alonso GAM,et al. Eur. Respir. J. 2020, 56 (suppl 64) 1776; doi: 10.1183/13993003.congress-2020.1776	No control group (without CAP). Only available in abstract.
	UK	Baskaran V, Mckeever T, Shen LW. Incidence of Cardiac Complications after Hospitalization for Community Acquired Pneumonia: A Large Population-based Cohort Study. Eur. Respir. J. 2020; 56: 1772 doi: 10.1183/13993003.congress-2020.1772	No control group (without CAP). Only available in abstract.

	China	Kang Y, Fang XY, Wang D, Wang XJ. Factors associated with acute myocardial infarction in older patients after hospitalization with community-acquired pneumonia: a cross-sectional study. BMC Geriatr. 2021;21(1):113. doi: 10.1186/s12877-021-02056-6.	No control group (without CAP).
	Italy	Pieralli F, Vannucchi V, Nozzoli C, Augello G, Dentali F, De Marzi G, et al. Acute cardiovascular events in patients with community acquired pneumonia: results from the observational prospective FADOI-ICECAP study. BMC Infect Dis. 2021 Jan 25;21(1):116. doi: 10.1186/s12879-021-05781-w.	No control group (without CAP).
	China	Huang X, Zhang J, Huang X, Zeng M, Chen Y. Predictors of 30-day mortality in elderly patients with community acquired pneumonia. Eur. Respir. J. 2015 46: PA2571; doi: 10.1183/13993003.congress-2015.PA2571	No control group (without CAP). Only available in abstract.
	Spain	Cilloniz CC, Liapikou A, Martin-Loeches I, Garcia-Vidal C, Gabarrus A, Ceccato A, et al. Twenty-year Trend in Mortality among Hospitalized Patients with Pneumococcal Community-Acquired Pneumonia. Eur. Respir. J. 2018 52: PA2626; doi: 10.1183/13993003.congress-2018.PA2626	No control group (without CAP). Only available in abstract.
	Zambia	Allen SC. Lobar pneumonia in Northern Zambia: clinical study of 502 adult patients. Thorax. 1984;39(8):612-6. doi: 10.1136/thx.39.8.612.	No control group (without CAP).
	USA	Esposito AL. Community-acquired bacteremic pneumococcal pneumonia. Effect of age on manifestations and outcome. Arch Intern Med. 1984;144(5):945-8. doi:10.1001/archinte.1984.00350170081016	No control group (without CAP).
	Canada	Marrie TJ, Durant H, Yates L. Community-acquired pneumonia requiring hospitalization: 5-year prospective study. Rev Infect Dis. 1989;11(4):586-99. doi: 10.1093/clinids/11.4.586.	No control group (without CAP).
	Sweden	Ortqvist A, Hedlund J, Grillner L, Jalonen E, Kallings I, Leinonen M, et al. Aetiology, outcome and prognostic factors in community-acquired pneumonia requiring hospitalization. Eur Respir J. 1990;3(10):1105-13.	No control group (without CAP).
	UK	Venkatesan P, Gladman J, Macfarlane JT, Barer D, Berman P, Kinnear W, et al. A hospital study of community acquired pneumonia in the elderly. Thorax. 1990;45(4):254-8. doi: 10.1136/thx.45.4.254.	No control group (without CAP).
	USA	Fine MJ, Smith DN, Singer DE. Hospitalization decision in patients with community-acquired pneumonia: a prospective cohort study. Am J Med. 1990;89(6):713-21. doi: 10.1016/0002-9343(90)90211-u.	No control group (without CAP).
	France	Leroy O, Santré C, Beuscart C, Georges H, Guery B, Jacquier JM, et al. A five-year study of severe community-acquired pneumonia with emphasis on prognosis in patients admitted to an intensive care unit. Intensive Care Med. 1995;21(1):24-31. doi: 10.1007/BF02425150.	No control group (without CAP).
	Switzerland	Janssens JP, Gauthey L, Herrmann F, Tkatch L, Michel JP. Community-acquired pneumonia in older patients. J Am Geriatr Soc. 1996;44(5):539-44. doi: 10.1111/j.1532-5415.1996.tb01439.x.	No control group (without CAP).

	UK	The aetiology, management and outcome of severe community-acquired pneumonia on the intensive care unit. The British Thoracic Society Research Committee and The Public Health Laboratory Service. Respir Med. 1992;86(1):7-13. doi: 10.1016/s0954-6111(06)80141-1.	No control group (without CAP).
	USA	Fine MJ, Stone RA, Singer DE, Coley CM, Marrie TJ, Lave JR, et al. Processes and outcomes of care for patients with community-acquired pneumonia: results from the Pneumonia Patient Outcomes Research Team (PORT) cohort study. Arch Intern Med. 1999;159(9):970-80. doi: 10.1001/archinte.159.9.970.	No control group (without CAP).
	USA	Musher DM, Alexandraki I, Graviss EA, Yanbeiy N, Eid A, Inderias LA, et al. Bacteremic and nonbacteremic pneumococcal pneumonia. A prospective study. Medicine (Baltimore). 2000;79(4):210-21. doi: 10.1097/00005792-200007000-00002.	No control group (without CAP).
	Spain	Fernández-Sabé N, Carratalà J, Rosón B, Dorca J, Verdaguer R, Manresa F, et al. Community-acquired pneumonia in very elderly patients: causative organisms, clinical characteristics, and outcomes. Medicine (Baltimore). 2003;82(3):159-69. doi: 10.1097/01.md.0000076005.64510.87.	No control group (without CAP).
	USA	Fine MJ, Stone RA, Lave JR, Hough LJ, Obrosky DS, Mor MK, et al. Implementation of an evidence-based guideline to reduce duration of intravenous antibiotic therapy and length of stay for patients hospitalized with community-acquired pneumonia: a randomized controlled trial. Am J Med. 2003;115(5):343-51. doi: 10.1016/s0002-9343(03)00395-4.	No control group (without CAP).
	Spain	Martínez-Moragón E, García Ferrer L, Serra Sanchis B, Fernández Fabrellas E, Gómez Belda A, Julve Pardo R. La neumonía adquirida en la comunidad de los ancianos: diferencias entre los que viven en residencias y en domicilios particulares [Community-acquired pneumonia among the elderly: differences between patients living at home and in nursing homes]. Arch Bronconeumol. 2004;40(12):547-52. Spanish. doi: 10.1016/s1579-2129(06)60373-x.	No control group (without CAP).
	Spain	Menéndez R, Torres A, Zalacaín R, Aspa J, Martín-Villasclaras JJ, Borderías L, et al. Guidelines for the treatment of community-acquired pneumonia: predictors of adherence and outcome. Am J Respir Crit Care Med. 2005;172(6):757-62. doi: 10.1164/rccm.200411-1444OC.	Clinical practice guideline.
	Spain	Querol-Ribelles JM, Tenías JM, Querol-Borrás JM, Labrador T, Nieto A, González-Granda D, et al. Levofloxacin versus ceftriaxone plus clarithromycin in the treatment of adults with community-acquired pneumonia requiring hospitalization. Int J Antimicrob Agents. 2005;25(1):75-83. doi: 10.1016/j.ijantimicag.2004.07.013.	No control group (without CAP).
	Chile	Díaz A, Alvarez M, Callejas C, Rosso R, Schnettler K, Saldías F. Cuadro clínico y factores pronósticos de la neumonía adquirida en la comunidad grave en adultos hospitalizados en la unidad de cuidados intensivos [Clinical picture and prognostic factors for severe community-acquired pneumonia in adults admitted to the intensive care unit]. Arch Bronconeumol. 2005;41(1):20-6. Spanish. doi: 10.1016/s1579-2129(06)60390-x.	No control group (without CAP).

	Canada	Marrie TJ, Huang JQ. Low-risk patients admitted with community-acquired pneumonia. Am J Med. 2005;118(12):1357-63. doi: 10.1016/j.amjmed.2005.06.035.	No control group (without CAP).
	Canada	McAlister FA, Majumdar SR, Blitz S, Rowe BH, Romney J, Marrie TJ. The relation between hyperglycemia and outcomes in 2,471 patients admitted to the hospital with community-acquired pneumonia. Diabetes Care. 2005;28(4):810-5. doi: 10.2337/diacare.28.4.810.	No control group (without CAP).
	Canada	Becker T, Moldoveanu A, Cukierman T, Gerstein HC. Clinical outcomes associated with the use of subcutaneous insulin-by-glucose sliding scales to manage hyperglycemia in hospitalized patients with pneumonia. Diabetes Res Clin Pract. 2007;78(3):392-7. doi: 10.1016/j.diabres.2007.05.003.	No control group (without CAP).
	Spain	Ramirez J, Aliberti S, Mirsaeidi M, Peyrani P, Filardo G, Amir A, et al. Acute myocardial infarction in hospitalized patients with community-acquired pneumonia. Clin Infect Dis. 2008;47(2):182-7. doi: 10.1086/589246.	No control group (without CAP).
	Spain	Cabré M, Serra-Prat M, Force L, Palomera E, Pallarés R. Functional status as a risk factor for mortality in very elderly patients with pneumonia. Med Clin (Barc). 2008;131(5):167-70. doi: 10.1157/13124262.	No control group (without CAP).
	Scotland	Mandal P, Chalmers JD, Choudhury G, Akram AR, Hill AT. Vascular complications are associated with poor outcome in community-acquired pneumonia. QJM. 2011;104(6):489-95. doi: 10.1093/qjmed/hcq247.	No control group (without CAP).
	USA	Perry TW, Pugh MJ, Waterer GW, Nakashima B, Orihuela CJ, Copeland LA, et al. Incidence of cardiovascular events after hospital admission for pneumonia. Am J Med. 2011;124(3):244-51. doi: 10.1016/j.amjmed.2010.11.014.	No control group (without CAP).
	Multicenter study (80 centers in 13 countries)	Griffin AT, Wiemken TL, Arnold FW. Risk factors for cardiovascular events in hospitalized patients with community-acquired pneumonia. Int J Infect Dis. 2013;17(12):e1125-9. doi: 10.1016/j.ijid.2013.07.005.	No control group (without CAP).
	Italy	Aliberti S, Ramirez J, Cosentini R, Valenti V, Voza A, Rossi P, et al. Acute myocardial infarction versus other cardiovascular events in community-acquired pneumonia. ERJ Open Res. 2015;1(1):00020-2015. doi: 10.1183/23120541.00020-2015.	No control group (without CAP).
	Italy	Cangemi R, Calvieri C, Falcone M, Bucci T, Bertazzoni G, Scarpellini MG, et al. Relation of Cardiac Complications in the Early Phase of Community-Acquired Pneumonia to Long-Term Mortality and Cardiovascular Events. Relation of Cardiac Complications in the Early Phase of Community-Acquired Pneumonia to Long-Term Mortality and Cardiovascular Events. Am J Cardiol. 2015;116(4):647-51. doi: 10.1016/j.amjcard.2015.05.028.	No control group (without CAP).
	Taiwan	Chen PC, Liao WI, Wang YC, Chang WC, Hsu CW, Chen YH, et al. An Elevated Glycemic Gap is Associated With Adverse Outcomes in Diabetic Patients With Community-Acquired Pneumonia. Medicine (Baltimore). 2015;94(34):e1456. doi: 10.1097/MD.0000000000001456.	No control group (without CAP).

	Italy	Violi F, Cangemi R, Falcone M, Taliani G, Pieralli F, Vannucchi V, et al. Cardiovascular Complications and Short-term Mortality Risk in Community-Acquired Pneumonia. Clin Infect Dis. 2017;64(11):1486-1493. doi: 10.1093/cid/cix164.	No control group (without CAP).
	Canada	Eurich DT, Marrie TJ, Minhas-Sandhu JK, Majumdar SR. Risk of heart failure after community acquired pneumonia: prospective controlled study with 10 years of follow-up. BMJ. 2017;356:j413. doi: 10.1136/bmj.j413.	No control group (without CAP).
	Turkey	Cilli A, Cakin O, Aksoy E, Kargin F, Adiguzel N, Karakurt Z, at al. Acute cardiac events in severe community-acquired pneumonia: A multicenter study. Clin Respir J. 2018;12(7):2212-2219. doi: 10.1111/crj.12791.	No control group (without CAP).
	Netherlands	Postma DF, Spitoni C, van Werkhoven CH, van Elden LJR, Oosterheert JJ, Bonten MJM. Cardiac events after macrolides or fluoroquinolones in patients hospitalized for community-acquired pneumonia: post-hoc analysis of a cluster-randomized trial. BMC Infect Dis. 2019;19(1):17. doi: 10.1186/s12879-018-3630-7.	No control group (without CAP).
	Italy	Spannella F, Giulietti F, Pimpini L, Lombardi FE, Re S, Schiavi P, et al. Performance of the CHA2DS2-VASc score in predicting new onset atrial fibrillation during hospitalization for community-acquired pneumonia. Eur J Intern Med. 2019;62:24-28. doi: 10.1016/j.ejim.2019.01.012.	No control group (without CAP).
	Italy	Cangemi R, Calvieri C, Taliani G, Pignatelli P, Morelli S, Falcone M, et al. Left Atrium Dilatation and Left Ventricular Hypertrophy Predispose to Atrial Fibrillation in Patients With Community-Acquired Pneumonia. Am J Cardiol. 2019;124(5):723-728. doi: 10.1016/j.amjcard.2019.05.051.	No control group (without CAP).
	Canada	Corrales-Medina VF, Taljaard M, Yende S, Kronmal R, Dwivedi G, Newman AB, et al. Intermediate and long-term risk of new-onset heart failure after hospitalization for pneumonia in elderly adults. Am Heart J. 2015;170(2):306-12. doi: 10.1016/j.ahj.2015.04.028.	No control group (without CAP).
	Canada.	Corrales-Medina VF, Taljaard M, Fine MJ, Dwivedi G, Perry JJ, Musher DM, et al. Risk stratification for cardiac complications in patients hospitalized for community-acquired pneumonia. Mayo Clin Proc. 2014;89(1):60-8. doi: 10.1016/j.mayocp.2013.09.015.	No control group (without CAP).
	Portugal	Fonseca A, Sá Marques M, Silva E, Shiang T, Vanzeller M, Ribeiro C. Community acquired pneumonia: an increased risk for subsequent cardiovascular events? Eur. Respir. J. 2020 56: 1775 doi: 10.1183/13993003.congress-2020.1775	No control group (without CAP). Only available in abstract.
